# Characterization of Antimicrobial Resistance and Hypervirulent Traits of *Klebsiella variicola* Isolates Collected in South Korea

**DOI:** 10.3390/antibiotics14101046

**Published:** 2025-10-18

**Authors:** Dokun Lee, Dokyun Kim, Hye Gyung Bae, Won-Jong Jang, Seok Hoon Jeong, Kyungwon Lee

**Affiliations:** 1 Department of Microbiology, College of Medicine, Konkuk University, Seoul 05029, Republic of Korea; 2 Department of Laboratory Medicine and Research Institute of Bacterial Resistance, Yonsei University College of Medicine, Seoul 06273, Republic of Korea; 3 Infectious Disease Research Center, Seoul Clinical Laboratories, Yongin 16954, Republic of Korea

**Keywords:** *Klebsiella variicola*, carbapenemase, virulence, whole-genome sequencing, South Korea

## Abstract

**Introduction**: *Klebsiella variicola*, a member of *Klebsiella pneumoniae* complex, has emerged as an opportunistic pathogen for human infection; however, antimicrobial resistance and hypervirulent characteristics of *K. variicola* have rarely been investigated in South Korea. **Methods**: We analyzed 76 clinical *K. variicola* isolates collected from 12 hospitals between September 2022 and October 2023. Bacterial identification was performed by MALDI-TOF MS. Antimicrobial susceptibility was tested by disk diffusion tests. Resistance determinants and virulence traits were investigated, and whole-genome sequencing was performed for hypermucoviscous or carbapenem-resistant *K. variicola* isolates. **Results**: Most (89.5%, 68/76) were susceptible to all 18 antimicrobials tested in this study, and 3 isolates harbored *bla*_CTX-M-15_. One isolate carried *bla*_KPC-2_ on its IncX3 plasmid, which is closely related to carbapenem-resistant *K. pneumoniae* plasmids. Capsular typing revealed 51 *wzi* allelic types. Ten isolates showed mucoid phenotype, mainly with KL60 and KL61. **Conclusions**: This study reveals relatively low resistance rates in *K. variicola* strains but the presence of multidrug-resistant and hypermucoviscous *K. variicola* strains. In addition, the evidence of interspecies dissemination of *bla*_KPC-2_ highlights the need for continuous genomic surveillance.

## 1. Introduction

The *Klebsiella pneumoniae* complex includes several species including *K. pneumoniae*, *K. quasipneumoniae*, *K. variicola*, *K. qusivariicola*, and *K. africanensis*. *K. pneumoniae* is one of the most important pathogens for both community-associated and hospital-originated infections, and emergence of hypervirulent (HV) and multidrug resistant (MDR) *K. pneumoniae* strains has been reported worldwide [1,2]. Furthermore, an outbreak of carbapenem-resistant and hypervirulent *K. pneumoniae* strains carrying a pLVPK-like virulence plasmid has been reported in China, which resulted in a fatal outcome for the infected patients [3].

*K. variicola* is another member of the *Klebsiella pneumoniae* complex, which was previously identified as the KpIII phylogroup of *K. pneumoniae* [4]. *K. variicola* exhibits biochemical characteristics similar to those of the other *K. pneumoniae* complex members, including positivity for urease, ortho-nitrophenyl-beta-galactoside, and the Voges-Proskauer test, while being negative for indole and ornithine decarboxylase [5], which causes misidentification among the *K. pneumoniae* complex members. For the accurate identification of *K. variicola* isolates, various approaches, including typing of chromosomal intrinsic β-lactamase, housekeeping gene analysis, single-nucleotide polymorphism-based average nucleotide identity analysis, and proteomic assay have been suggested [4], and Matrix-Assisted Laser Desorption Ionization Time-of-Flight Mass Spectrometry (MALDI-TOF MS) showed reliable discrimination accuracy among the *K. pneumoniae* complex members [6].

*K. variicola* causes various kinds of human infections, including bloodstream infection, urinary tract infection, and pneumonia [7]. Considering the previous misidentification of *K. variicola* to *K. pneumoniae* in the era before MALDI-TOF MS application, the public burden of *K. variicola* has been underscored [8]. Recently, extended-spectrum β-lactamase (ESBL-)-producing *K. variicola* have been reported in the US, Norway, and China [3,9,10]. Furthermore, carbapenemase-producing *K. variicola* strains including KPC-2, NDM-1 and NDM-5 have been identified [11]. Similarly to *K. pneumoniae*, hypervirulent *K. variicola* strains with ESBL and carbapenemase-production also emerged in China [12]. Hypervirulent *K. pneumoniae* complex has been associated invasive liver abscess syndrome, and poor clinical outcome has been reported when accompanied with multidrug resistance [13]. Despite the increasing concern about the dissemination of hypervirulent and MDR *K. variicola* strains, molecular epidemiologic research about *K. variicola* isolates in South Korea is limited to date.

The aim of this study is to characterize the antimicrobial susceptibility profile, related resistance determinants, and the virulence factors of *K. variicola* collected in South Korea.

## 2. Results

### 2.1. Collection of K. variicola Isolates

**Figure 1:**
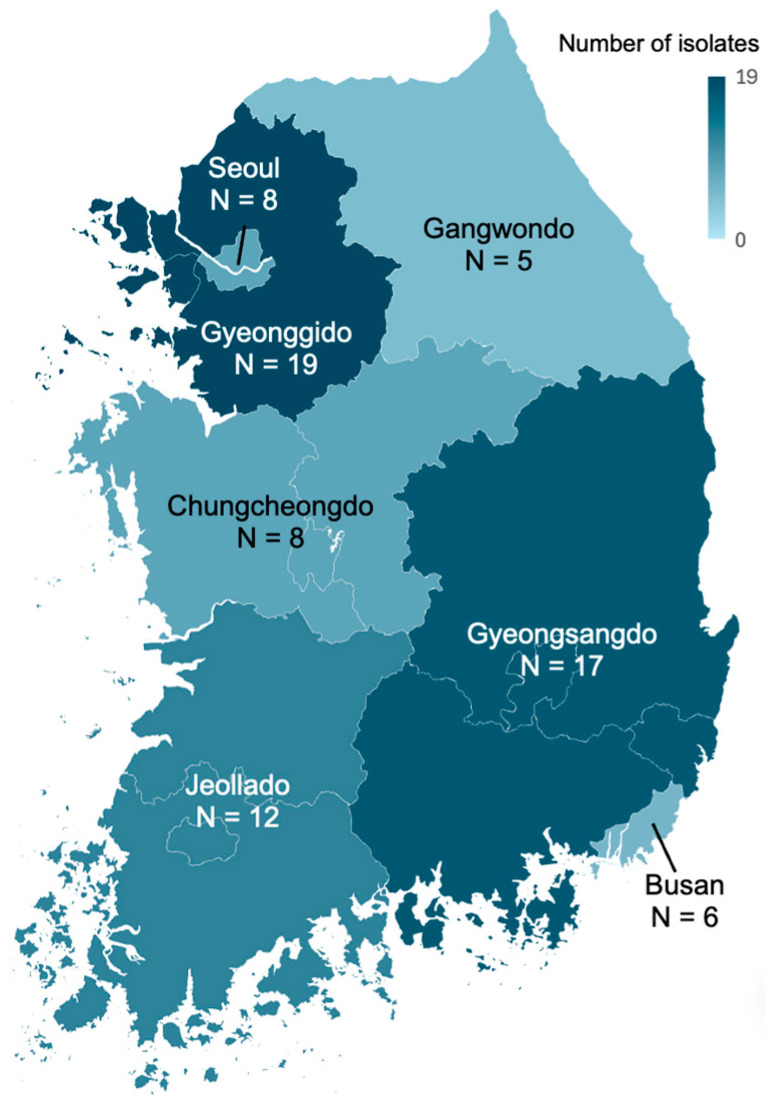
The geographic distribution of *K. variicola* isolates.

During the study period, a total of 76 *K. variicola* isolates were collected (Table 1 and Figure 1). About three-fourths of isolates (72.4%, *n* = 55) were collected from female patients, and 69.7% (*n* = 53) were collected from elderly patients over 60. The most common specimen type was urine (52.6%, *n* = 46), followed by abscess (22.4%, *n* = 17), blood (7.9%, *n* = 6), and sputum (6.6%, *n* = 5).

### 2.2. Antimicrobial Resistance Profiles and β-Lactamase Genotype of K. variicola Isolates

**Figure 2:**
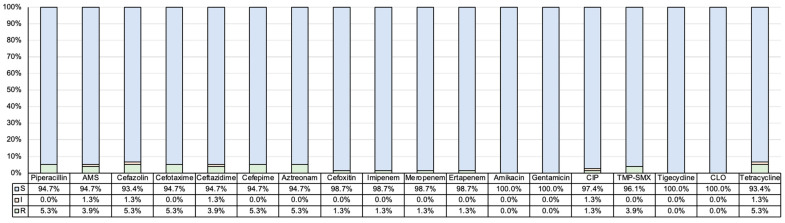
Antimicrobial resistance rates of *K. variicola* isolates. Abbreviation: AMS, ampicillin-sulbactam; CIP, ciprofloxacin; TMP-SMX, trimethoprim-sulfamethoxazole; CLO, chloramphenicol.

The resistance rate of *K. variicola* isolates was less than 10% in all antimicrobials tested in this study (Figure 2). Among the 76 *K. variicola* clinical isolates, 68 isolates (89.5%) exhibited susceptibility to all 18 antimicrobials. Only four isolates (5.3%) showed nonsusceptibility to the third-generation cephalosporins, including both cefotaxime and ceftazidime, and one of them showed carbapenem resistance.

The chromosomal SHV-OKP-LEN β-lactamase genotyping revealed that most of *K. variicola* isolates possessed *bla*_LEN_, except for one isolate (KV29) which carried *bla*_SHV-11_. Among the 76 *bla*_LEN_-possessing *K. variicola* isolates, 26 distinct LEN genotypes, including 13 novel allelic types, were identified. The most common LEN genotype was LEN-13 (*n* = 11), followed by LEN-New1 (*n* = 8), LEN-27 (*n* = 7), and LEN-16, LEN-New2, and LEN-New10 (*n* = 6 each).

**Figure 3:**
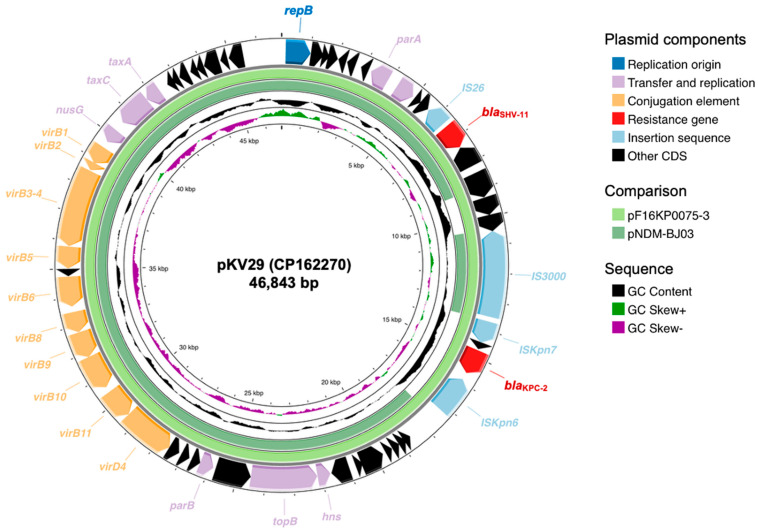
Schematic map of pKV29. From the outside, the outermost circle presents the open reading frames of pKV29. The colors of arrows were classified according to the function of each gene, including blue for replication origin, light purple for transfer and replication, yellow for conjugation, red for resistance, and light blue for insertion sequences. The inner two circles with emerald and light green indicate the regions that showed homology (>99% nucleotide similarity) with the plasmids published previously (pF16KP0075-3, GenBank accession: CP052170.1; pNDM-BJ03, GenBank accession: JX104760.1). The innermost circles present GC content and GC skew.

Three cefotaxime-resistant *K. variicola* harbored *bla*_CTX-M-15_, and one cefotaxime- and ertapenem-resistant isolate (KV29) possessed *bla*_KPC-2_ in its plasmid (Figure 3). This plasmid (pKV29, GenBank accession: CP162270) belonged to the IncX3 family plasmid with 46,843 bp, and harbored conjugative type IV secretion system gene clusters, and an additional β-lactamase gene, *bla*_SHV-11_. pKV29 showed only a 7 bp difference with pF16KP0075-3 (GenBank accession: CP052170) identified in carbapenem-resistant *K. pneumoniae* clinical isolates in Korea [14], and the core elements of the plasmid showed >99% similarity with pNDM-BJ03 (GenBank accession: JX104760.1) [15].

### 2.3. Capsular Type and Virulence Traits of K. variicola

**Figure 4:**
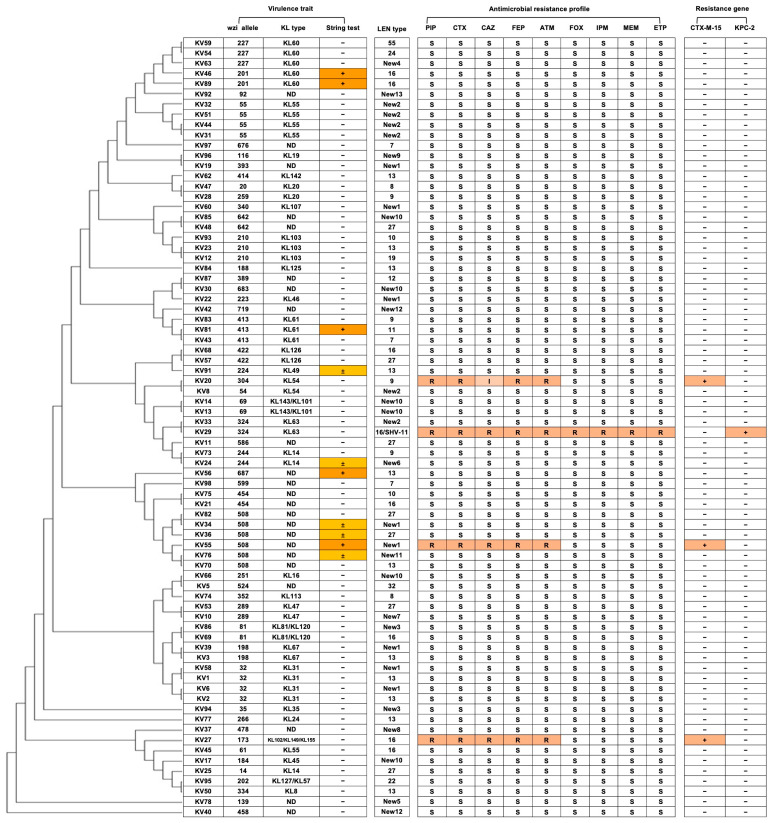
Phylogenetic tree of *K. variicola* isolates based on the sequences of the *wzi* gene. Positive reactions in the string test (+) are highlighted in orange, and weakly positive reactions (±) in light orange. Resistance to antimicrobials is shaded in apricot, with intermediate resistance shown in light apricot. The presence of resistance genes is also indicated in apricot. Abbreviation: PIP, piperacillin; CTX, cefotaxime; CAZ, ceftazidime; FEP, cefepime; ATM, aztreonam; FOX, cefoxitin; IPM, imipenem; MEM, meropenem; ERT, ertapenem; S, susceptible; I, intermediate; R, resistant; ND, not determined; New, New allele.

The capsular typing based on the *wzi* sequence revealed that 76 *K. variicola* isolates belonged to 51 different *wzi* allelic types (Figure 4). The most common capsular type was *wzi*508 (*n* = 6), followed by *wzi*32-KL31 (*n* = 4), *wzi*55-KL55 (*n* = 4), *wzi*210-KL103 (*n* = 3), *wzi*227-KL60 (*n* = 3), and *wzi*413-KL61 (*n* = 3). Ten *K. variicola* isolates exhibited weakly positive (*n* = 5) or positive (*n* = 5) reactions in the string test. Among the five string test-positive isolates, two belonged to *wzi*201-KL60, each one to *wzi*413-KL61, *wzi*687, and *wzi*508. Three other *wzi*508 isolates and each one of *wzi*224-KL49 and *wzi*244-KL14 exhibited weakly positive in the string tests. Most of hypermucoviscous isolates were susceptible to most of antimicrobials, and only one isolate (KV55) harbored the *bla*_CTX-M-15_ gene. Whole-genome sequencing revealed that all hypermucoviscous *K. variicola* isolates possessed virulence trait-related genes for iron acquisition (*kfu*), adhesin (*mrk*), and urease (*ureA*); however, common capsule production-associated genes such as *magA*, *rmpA*, or *rmpA2*, or salmochlelin/yersiniabactin-related gene including *iroB* or *irp2,* were not identified in any isolate. In addition, both *wzi*201-KL60 isolates possessed a IncF1B-type plasmid.

## 3. Discussion

*K. variicola* is a member of the *K. pneumoniae* complex, and automated bacterial identification systems have often caused misidentification as *K. pneumoniae* due to the similarity of biochemical reactions [8]. Whole-genome sequencing could be a standard for species-level discrimination within the *K. pneumoniae* complex, but its application in routine clinical microbiology remains limited. Through the clinical application of MALDI-TOF MS and the use of expanded reference databases, accurate species-level discrimination within the *K. pneumoniae* complex has become possible in clinical microbiology laboratories [6], and recent studies revealed that the actual prevalence of *K. variicola* infection had been underestimated [16]. Previous studies have suggested that the family of the chromosomal beta-lactamase genes could be genetic markers for species-level identification of the *K. pneumoniae* complex: *bla*_SHV_ for *K. pneumoniae*, *bla*_OKP_ for *K. quasipneumoniae*, and *bla*_LEN_ for *K. variicola* [4]; however, the acquisition of plasmid harboring *bla*_SHV_ could lead to misidentification, as seen in KV29 in this study.

Multidrug-resistant *K. variicola* isolates have been reported in China, Japan, European countries, and the USA [1,3,6,11], although those reports remain relatively rare. The first case of ESBL-producing *K. variicola* outbreak was identified in Mexico, of which the genotype of ESBL was SHV type [17]. The CTX-M-type ESBL-producing *K. variicola* has been identified in the US and Japan [10,16]. In our study, four isolates were identified as resistant to cefotaxime, and three of them were *bla*_CTX-M-15_-harboring *K. variicola* isolates, which indicates that the MDR *K. variicola* is still rare in South Korea and the ESBL genotype of *K. variicola* is still limited. The CTX-M-15 is the most common ESBL identified in *K. pneumoniae* isolates in Korea, accounting for approximately four-fifths of ESBL-producing *K. pneumoniae*, while *E. coli* shows a more diverse distribution of CTX-M genotypes [2]. The *bla*_CTX-M-15_-harboring *K. variicola* isolate, which possessed chromosomal *bla*_LEN-16_, was reported in the US, similar to the KV-27 strain in this study [10]. Carbapenemase-producing *K. variicola* is still rare, but KPC-2- or NDM-9-producing *K. variicola* strains have been reported in the US, China, India, and Chile, mostly related to ST69 and ST93 *K. variicola* strains [7]. Considering that *K. variicola* and *K. pneumoniae* belong to the same complex, it might be hypothesized that they could share a similar resistance gene profile or exhibit species-specific associations of mobile genetic elements at the transposon or plasmid level. One *bla*_KPC-2_-carrying isolate was identified in this study, and the plasmid structure was almost identical to that previously reported in the clinical *K. pneumoniae* strain collected from the patient with bloodstream infection in Korea [14]. These findings might suggest the potential for interspecies dissemination of resistance determinants within the *K. pneumoniae* complex.

Hypermucoviscous or hypervirulent traits of the *K. pneumoniae* complex have been increasingly recognized as an important pathogen for liver abscess syndrome [13]. The definition of hypervirulence in the *K. pneumoniae* complex has not been firmly established. However, several genetic biomarkers have been suggested, including capsule production-associated genes such as plasmid-borne *rmpA* and siderophore-related genes including *iroB* or *irp2* [18]. In addition, the mucoid phenotype is often regarded as a common characteristic of hypervirulent strains. While hypervirulent *K. pneumoniae* has been investigated broadly, the reports of hypervirulent *K. variicola* remain scarce. A hypermucoviscous *K. variicola* strain belonging to the KL114 capsular type harboring a 343 kb plasmid (pKV8917) was reported in 2020 [19], and carbapenem-resistant and hypervirulent *K. variicola* KL16 strains were identified in China [3]. In this study, the capsular typing revealed 51 different *wzi* allelic types, which suggests heterogenic distribution rather than the presence of predominant clone. Two *wzi*201-KL60 strains showed a hypermucoviscous phenotype, and three out of four *wzi*508 strains were weakly positive or positive in the string tests. The reports about *wzi*201-KL60 are very rare, and the KL60 strain has been reported to exhibit diverse structural (penta- to heptasaccharides) and biochemical capsule compositions, especially enriched in glucose [20], and a hypervirulent carbapenemase-producing KL60 *K. pneumoniae* clone has been introduced in China [3], while the *wzi*508 *K. variicola* strain was never been reported in data. Further surveillance and functional investigation of emerging clones with diverse virulence traits should be performed.

One limitation of this study is that this study was performed in a single country with a relatively short collection period, resulting in a limited number of multidrug-resistant and hypermucoviscous isolates. Therefore, the representativeness of global or national epidemiology could not be explored. Further investigations with larger numbers of *K. variicola* isolates and expanding the sampling to the hospital environment, livestock, or wastewater are needed to clarify the nationwide and long-term epidemiology of this species.

## 4. Materials and Methods

### 4.1. Bacterial Isolates

*K. variicola* isolates from September 2022 and October 2023 from 12 hospitals were collected from Seoul Clinical Laboratories. All *K. variicola* isolates collected from clinical specimens during the study period were included in this study, and duplicated isolates were excluded. The sentinel hospitals are located in the 8 different districts of South Korea.

### 4.2. Species Identification and Antimicrobial Susceptibility Tests

Bacterial identification was performed by Bruker Biotyper (Bruker Daltonics, Billerica, MA, USA). Briefly, pure colonies were applied to a metal plate, and a matrix solution was inoculated. The protein spectra according to *m*/*z* ratio were acquired and were compared to the database version 13. Antimicrobial susceptibility tests of *K. variicola* isolates were performed by disk diffusion methods on Mueller-Hinton agar (Difco Laboratories, Detroit, MI, USA) following the CLSI guideline [21], with the following antimicrobials: piperacillin, ampicillin–sulbactam, cefazolin, cefotaxime, ceftazidime, cefepime, aztreonam, cefoxitin, ertapenem, imipenem, meropenem, trimethoprim–sulfamethoxazole, tigecycline, amikacin, tetracycline, chloramphenicol, ciprofloxacin, and gentamicin. Escherichia coli ATCC 25922 was used as quality control.

### 4.3. β-Lactamase Genotype

The chromosomal SHV-OKP-LEN β-lactamase genotyping was performed in all *K. variicola* isolates by PCR and sequencing with the primers (forward: 5′-CCGGGTTATTCTTATTTGTCGCT-3′; reverse: 5′-TAGCGTTGCCAGTGCTCG-3′) previously described [22]. PCR was carried out under the following conditions: initial denaturation at 94 °C for 5 min; 35 cycles of 94 °C for 30 s, 61 °C for 30 s, and 72 °C for 30 s; followed by a final extension at 72 °C for 7 min. Amplicons were sequenced by the same primers. Putative ESBL or carbapenemase producers were selected based on the resistance profile of beta-lactam antimicrobials according to the EUCAST guideline [23]. PCR and sequencing were performed for the determination of ESBL genotypes for CTX-M type and SHV, and carbapenemase genotypes for KPC, NDM, VIM, IMP, and OXA-48. The genotypes were confirmed by comparing them to the Beta-Lactamase Database (last updated on 29 March 2025; http://www.bldb.eu).

### 4.4. Determination of Hypervirulent Trait

String test was performed to identify the hypermucoviscous phenotype of *K. variicola* isolates, which is one of the key indicators of hypervirulent strains. Briefly, *K. variicola* isolates were inoculated on a blood agar plate (Synergy Innovation Co., Gyeonggido, Republic of Korea) and incubated overnight at 37 °C. Bacterial colonies were touched and gently pulled upward with inoculation loops. The formation of a viscous string measuring 3–5 mm was interpreted as weakly positive, and a string longer than 5 mm was considered positive. A hypervirulent *K. pneumoniae* strain identified as K1 capsular type in our previous study was used as a positive control [24]. For the investigation of the capsular types, PCR and sequencing of the *wzi* gene was performed with the primers (forward: 5′-GTGCCGCGAGCGCTTTCTATCTTGGTATTCC-3′; reverse: 5′-GAGAGCCACTGGTTCCAGAAYTTSACCGC-3′). The PCR conditions of *wzi* were as follows: initial denaturation at 94 °C for 5 min; 30 cycles of denaturation at 94 °C for 30 s, annealing at 55 °C for 40 s, and extension at 72 °C for 30 s; and final extension at 72 °C for 5 min. The *wzi* allelic types of *K. variicola* isolates were identified by comparing the 447 bp partial sequences of the *wzi* gene against the *K. pneumoniae wzi* database (http://bigsdb.pasteur.fr/klebsiella/, accessed on 16 September 2025). Based on the wzi allelic types, capsular type (KL type) was determined, and unassigned allelic types were designated as ‘not determined (ND)’.

### 4.5. Whole-Genome Sequencing of K. variicola Isolates

Microbial whole-genome sequence (WGS) analysis was performed for hypermucoviscous *K. variicola* isolates exhibiting weakly positive or positive in the string tests, and one carbapenem-resistant isolate. Genomic DNA of *K. variicola* isolates was extracted using MagMAX^TM^ Microbiome Ultra Nucleic Acid Isolation Kit (Thermo Fisher Scientifics, Waltham, MA, USA), following the manufacturer’s instructions. Briefly, 200 μL of bacterial suspension was mixed with 800 μL of lysis buffer and vortexed at 2500 rpm. Lysed samples were then vortexed for 10 min, and centrifuged at 14,000× *g* for 2 min. Subsequently, 40 μL of proteinase K was added, and the mixture was incubated for 20 min. After followed the four washing steps: two with wash buffer and RNase 10 μL, and two with 80% ethanol. Finally, extracted DNA was eluted in 80 μL of elution solution. The concentration of extracted DNA was measured by Qubit fluorometer (Thermo Fisher Scientifics). Libraries were prepared with extracted DNA using SMRTbell Express Template Prep Kit 3.0 (Pacific Biosciences of California, Menlo Park, CA, USA). Briefly, repair and A-tailing were performed with 46 μL of extracted DNA and 14 μL of Repair master mix, and adapter ligation was performed at 20 for 30 min with 4 μL of adapter mix, 30 μL of ligation mix, and 1 μL of ligation enhancer, followed by nuclease treatment and additional bead purification. Microbial genome was sequenced with a SMRT cell 1 M on the PacBio Sequel II system (Pacific Biosciences of California, Menlo Park, CA, USA). Raw sequence reads were assembled using PBMM2 (https://github.com/PacificBiosciences/pbmm2, last updated in February 2025, accessed on 16 September 2025), and the assembled contigs were annotated by PROKKA [25].

### 4.6. In Silico Analysis of Whole Genome Sequencing Data

Multilocus sequence typing (MLST) and core genome (cgMLST) of the WGS data were determined by Ridom SeqSphere version 9.0.3. Briefly, allelic types of nine housekeeping genes of the MLST scheme and 2358 core genome of the cgMLST scheme were identified using fastq files of the bacterial whole genome, and sequence type (ST) and clonal group were determined based on the allelic profiles. Virulence factors including *kfuA*, *kfuB*, *kfuC*, *entB*, *cf29a*, *fimH*, *mrkD*, *pks*, *ureA*, and *allS* were identified by comparing with the Institut Pasteur database (http://bigsdb.pasteur.fr, accessed on 16 September 2025). The plasmid replicon genes were identified by Plasmid Finder using the fastq file of the bacterial whole genome, with the database updated in January 2023 (https://cge.food.dtu.dk/services/PlasmidFinder/, accessed on 16 September 2025) [26]. The NCBI Basic Local Alignment Search Tool was used to compare the structure of plasmids, and the plasmid map was visualized using the Proksee online tool (https://proksee.ca/) [27].

### 4.7. Statistical Analysis

The comparisons between the groups were performed by Fisher’s exact test in categorical variables, and by the Mann–Whitney U test in continuous variables. All statistical analyses were performed using R software version 4.3.1 with the package ‘moonbook’.

## 5. Conclusions

This study presents the antimicrobial resistance and virulence traits of clinical *K. variicola* isolates in South Korea, suggesting the emergence of multidrug-resistant *K. variicola* strains harboring *bla*_CTX-M-15_ and *bla*_KPC-2_, and hypermucoviscous strains, such as *wzi*201-KL60 and *wzi*501 strains. Although most *K. variicola* isolates remained susceptible to most antimicrobials, the interspecies dissemination of resistance determinants within the *K. pneumoniae* complex highlights a potential threat for further dissemination. Continuous molecular surveillance is needed to understand the clinical impact and evolutionary dynamics of *K. variicola*.

## Figures and Tables

**Table 1:** Clinical characteristics of patients with *K. variicola* infection.

Variable	Total (*n* = 76)	Male (*n* = 21)	Female (*n* = 55)	*p*-Value
Old age (>60)	53 (69.7)	17 (81.0)	34 (61.8)	0.189
Regions				0.432
Gyeonggido	19 (25)	4 (19.0)	15 (27.3)	
Gyeongsangdo	17 (22.4)	3 (14.3)	14 (25.5)	
Jeollado	12 (15.8)	4 (19.0)	8 (14.5)	
Chungcheongdo	8 (10.5)	2 (9.5)	6 (10.9)	
Seoul	8 (10.5)	5 (23.8)	3 (5.5)	
Busan	6 (7.9)	2 (9.5)	4 (7.3)	
Gangwondo	5 (6.6)	1 (4.8)	4 (7.3)	
Type of hospital				0.032
General hospital	42 (55.3)	16 (76.2)	26 (47.3)	
Clinic	20 (26.3)	2 (9.5)	18 (32.7)	
Nursing hospital	4 (5.3)	1 (4.8)	3 (5.5)	
Others	10 (13.2)	2 (9.5)	8 (14.5)	
Specimen				<0.001
Urine	40 (52.6)	3 (14.3)	37 (67.3)	
Abscess	17 (22.4)	8 (28.6)	9 (16.4)	
Blood	6 (7.9)	5 (23.8)	1 (1.8)	
Sputum	5 (6.6)	4 (19.0)	1 (1.8)	
Others	8 (10.5)	1 (4.8)	7 (12.7)	

## Data Availability

The microbial whole-genome data of bacterial strains are available in the National Center for Biotechnology Informatics in BioProject under accession PRJNA1131943.
